# Supplementary material for: Experimental observation of flow fields around active Janus spheres
Source: Nat Commun. 2019 Sep 2;10:3952. doi: 10.1038/s41467-019-11842-1 (PMC6718378; doi:10.1038/s41467-019-11842-1)
Supplement: Supplementary file 1 — Supplementary Information [file 41467_2019_11842_MOESM1_ESM.pdf]

Experimental observation of flow fields around active Janus spheres
Supplementary Information

Andrew I. Campbell,[1] Stephen J. Ebbens,[1,*] Pierre Illien,[2] and Ramin Golestanian[3,4,†]

[1]*Department of Chemical and Biological Engineering, University of Sheffield Mappin Street, Sheffield S1 3JD, UK*

[2]*Sorbonne Université, CNRS, Laboratoire PHENIX, 4 place Jussieu, 75005 Paris, France*

[3]*Max Planck Institute for Dynamics and Self-Organization (MPIDS), 37077 Göttingen, Germany*

[4]*Rudolf Peierls Centre for Theoretical Physics, University of Oxford, Oxford OX1 3PU, United Kingdom*

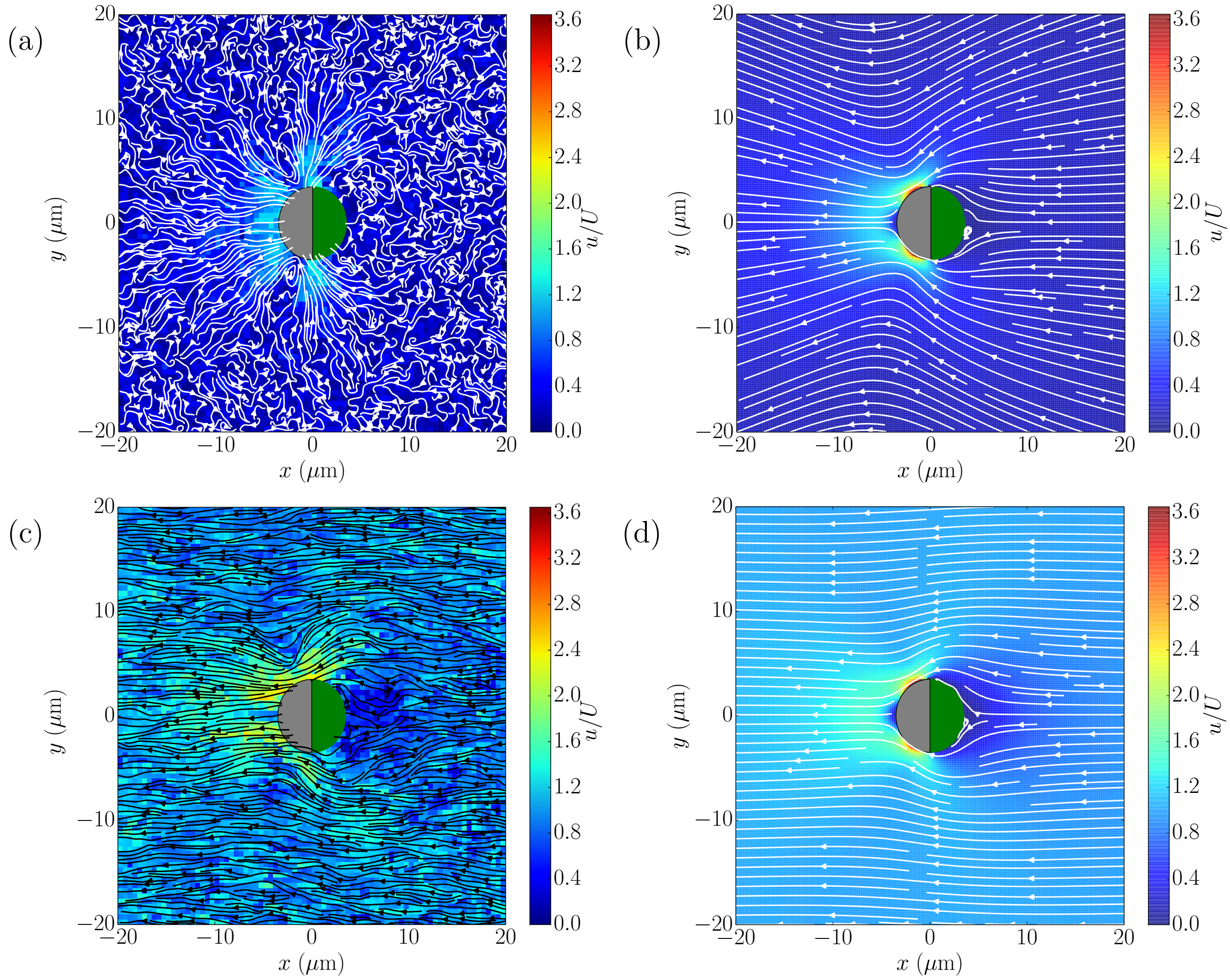

Supplementary Figure 1: Zoomed-out version of Fig. 1 (c)-(d) from the main text. (a)-(d) Streamlines around the Janus particles obtained experimentally (left) and analytically (right), in the two situations where the particle is stuck (top) and freely moving (bottom). The background colors represent the magnitude of the velocity $u = |\boldsymbol{u}|$ rescaled by the swimming velocity of the Janus particle when it is freely moving.

*Electronic address: `s.ebbens@sheffield.ac.uk`

†Electronic address: `ramin.golestanian@ds.mpg.de`

Supplementary Note 1: Consistency between the measurements of the flow fields around a stuck colloid and around a moving colloid

The velocity field around a stuck colloid can be related to the velocity field around a moving colloid through

$$\boldsymbol{u}^{\text{stuck}} = \boldsymbol{u}^{\text{moving}} + U\hat{\boldsymbol{e}} + \boldsymbol{u}^{\text{monopole}} \tag{1}$$

where $\boldsymbol{u}^{\text{stuck}}$ is given by Eq. (15) from the main text, $\boldsymbol{u}^{\text{moving}}$ is given by Eq. (17) from the main text, and $\boldsymbol{u}^{\text{mono}}$ is given by Eq. (10) from the main text. In order to highlight the consistency between the measurements in the stuck and in the freely swimming cases, we compute the quantity $\delta\boldsymbol{u} = \boldsymbol{u}^{\text{stuck}} - \boldsymbol{u}^{\text{moving}} - \boldsymbol{u}^{\text{monopole}}$. The value of the force is chosen such that $f = 6\pi\eta a U$ (see main text). We present on Supplementary Figure 2 the vector field $\delta\boldsymbol{u}$. As $\delta\boldsymbol{u}$ is expected to be equal to $U\hat{\boldsymbol{e}}$ anywhere around the colloid, we compute $\langle\delta u_x\rangle$ and $\langle\delta u_y\rangle$, where the averages run over all measurement points, and find

$$\langle\delta u_x\rangle = (1.14 \pm 0.29)U, \tag{2}$$
$$\langle\delta u_y\rangle = (-0.0011 \pm 0.28)U, \tag{3}$$

which is a strong indication that Eq. (1) holds experimentally, and which highlights the consistency between the two sets of experimental measurements.

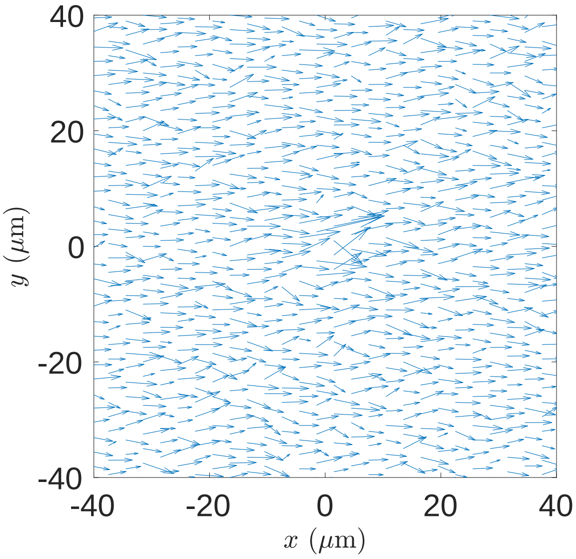

Supplementary Figure 2: Vector plot of $\delta\boldsymbol{u}$ as defined in Eq. (1).

Supplementary Note 2: Influence of the solid wall

In this section, the origin of coordinates is a point on the solid wall (see Supplementary Figure 3). Following Spagnolie and Lauga [1], the flow field created by a squirmer whose centre is at position $\boldsymbol{r}_0 = (0, 0, h)$ and with zero rotational velocity can be decomposed into a sum of singularities which correspond to a far field expansion ([1] Eq. (2.17)):

$$\boxed{\widetilde{\boldsymbol{u}}_{\text{sing,free}}(\boldsymbol{r}) = -\hat{\boldsymbol{e}} + \alpha \boldsymbol{G_D}(\boldsymbol{r}-\boldsymbol{r}_0, \hat{\boldsymbol{e}}, \hat{\boldsymbol{e}}) + \beta a \boldsymbol{D}(\boldsymbol{r}-\boldsymbol{r}_0, \hat{\boldsymbol{e}}) + \gamma a \boldsymbol{G_Q}(\boldsymbol{r}-\boldsymbol{r}_0, \hat{\boldsymbol{e}}, \hat{\boldsymbol{e}}) + \mathcal{O}(|\boldsymbol{r}-\boldsymbol{r}_0|^{-4})} \tag{4}$$

(note that this is dimensionless i.e. the velocity is measured in units of $2B_1/3$ and Lauga's result was modified to be in the frame of reference attached to the colloid). Here α, β and γ are dimensionless coefficients that characterize the strength of each singularity, and one defines:

- the Stokeslet dipole $\boldsymbol{G_D}$, see [1] Eq. (A2),
- the source dipole $\boldsymbol{D}$, see [1] Eq. (A8),
- the Stokeslet quadrupole $\boldsymbol{G_Q}$ [1] Eq. (A3).

Ishimoto and Gaffney [2] give the relation between α, β and γ and the Legendre coefficients of the slip velocity at the surface of the colloid (see [2] Eq. (7)):

$$\alpha = -\frac{3}{4}\frac{B_2}{B_1} \tag{5}$$

$$\beta = \frac{1}{2} - \frac{1}{8}\frac{B_3}{B_2} \tag{6}$$

$$\gamma = -\frac{5}{16}\frac{B_3}{B_2} \tag{7}$$

They are indeed dimensionless since each of the B_n has the dimension of a velocity. We note that because it relies on a singularity expansion, the expression of $\widetilde{\boldsymbol{u}}$ in Eq. (4) only involves the first three Legendre coefficients of the slip velocity B_1, B_2 and B_3. Any higher-order contribution to the slip velocity won't have any effect on the computed $\widetilde{\boldsymbol{u}}$ at this level of expansion.

Applying the method of images, the flow field in the presence of the wall is obtained from $\widetilde{\boldsymbol{u}}$ in Eq. (4) by adding to each of the singularities its image:

$$\boxed{\begin{aligned}\widetilde{\boldsymbol{u}}_{\text{sing,wall}}(\boldsymbol{r}) = -\hat{\boldsymbol{e}} &+ \alpha \boldsymbol{G_D}(\boldsymbol{r}-\boldsymbol{r}_0, \hat{\boldsymbol{e}}, \hat{\boldsymbol{e}}) + \beta a \boldsymbol{D}(\boldsymbol{r}-\boldsymbol{r}_0, \hat{\boldsymbol{e}}) + \gamma a \boldsymbol{G_Q}(\boldsymbol{r}-\boldsymbol{r}_0, \hat{\boldsymbol{e}}, \hat{\boldsymbol{e}}) \\ &+ \alpha \boldsymbol{G}^*_{\boldsymbol{D}}(\boldsymbol{r}-\boldsymbol{r}^*_0, \hat{\boldsymbol{e}}, \hat{\boldsymbol{e}}) + \beta a \boldsymbol{D}^*(\boldsymbol{r}-\boldsymbol{r}^*_0, \hat{\boldsymbol{e}}) + \gamma a \boldsymbol{G}^*_{\boldsymbol{Q}}(\boldsymbol{r}-\boldsymbol{r}^*_0, \hat{\boldsymbol{e}}, \hat{\boldsymbol{e}}) + \mathcal{O}(|\boldsymbol{r}-\boldsymbol{r}_0|^{-4}),\end{aligned}} \tag{8}$$

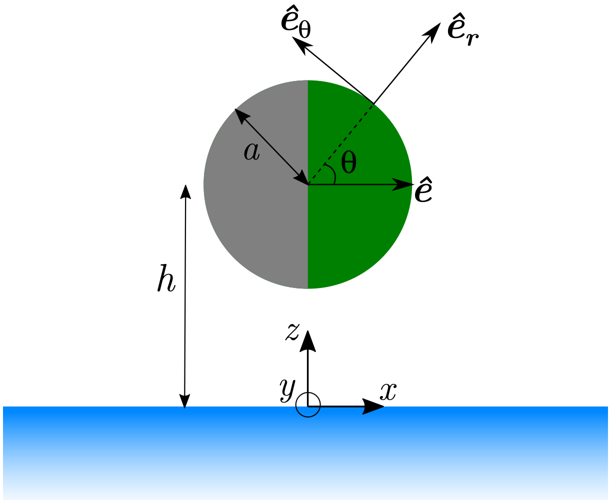

Supplementary Figure 3: Notations for the singularities calculation

where $\boldsymbol{r}_0^* = (0, 0, -h)$ is the image of the singularity located at $\boldsymbol{r}_0$. Although the image of a Stokeslet has a simple expression and was computed long ago (see e.g. [3]), the image of the singularities present in Eq. (4) (Stokeslet dipole, source dipole, Stokeslet quadrupole) have complicated expressions but are given in [1]:

- the image of the Stokeslet dipole $\boldsymbol{G}_{\boldsymbol{D}}^*$ is given in [1] Eq. (B5),
- the image of the source dipole $\boldsymbol{D}^*$ is given in [1] Eq. (B13),
- the image of the Stokeslet quadrupole $\boldsymbol{G}_{\boldsymbol{Q}}^*$ is given in [1] Eq. (B8).

In the main text, as examples of the influence of the solid wall on the calculated flow field, we plot on Fig. 5 two components of the flow field around the moving swimmer using the three different analytical expressions, obtained respectively with the singularities approximation in free space [Eq. (4)], the singularities approximation with the influence of the wall [Eq. (8)] and the exact solution in free space valid at any point (see Methods section).

Supplementary Note 4: Flow fields generated by alternative simple slip velocity profiles

Slip velocity with a dipolar symmetry

The flow field generated by the nonzero slip velocity at the surface of the colloid can again be calculated Eqs. (15) and (17) from the main text, but with another expression for the slip velocity at the surface of the colloid $v(\theta)$.

We first consider a slip velocity with a dipolar symmetry, that is peaked at the equator of the particle and vanishes at its poles:

$$v(\theta) = v_0 \sin\theta \tag{9}$$

The velocity profiles $u_x(x, y = 0, z = 0)$ and $u_y(x = 0, y, z = 0)$ can be calculated and fitted to the experimental measurements using v_0 as a fit parameter (Supplementary Figure 4). The results are commented in the main text.

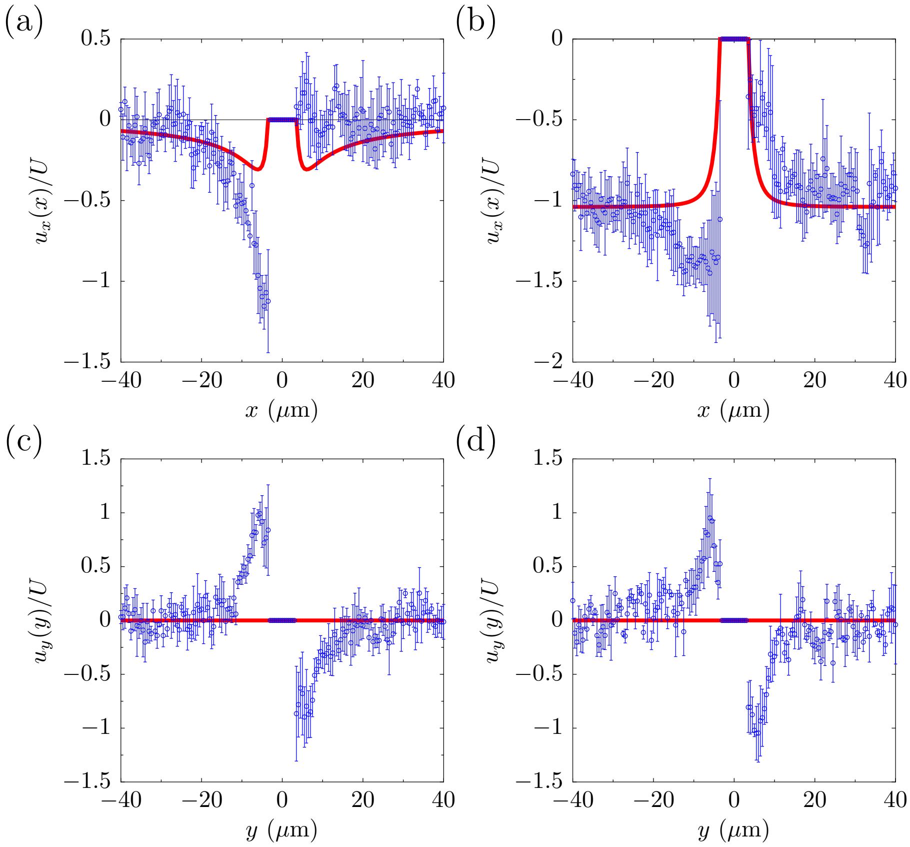

Supplementary Figure 4: Top: x-component of the velocity measured at $y = 0$ and as a function of the coordinate x for the two situations where the swimmer is stuck (a) and freely moving (b). Bottom: y-component of the velocity measured at $x = 0$ and as a function of the coordinate y for the two situations where the swimmer is stuck (c) and freely moving (d). The red line is a fit of the experimental data using a slip velocity with dipolar symmetry [Eq. (9)]. The values of the fit parameters are $K_{\text{stuck}} = 0.80 \pm 0.26$ and $K_{\text{moving}} = 1.56 \pm 0.34$.

Constant slip velocity over the Pt hemisphere

We then consider a slip velocity that is constant over the Pt hemisphere:

$$v(\theta) = \begin{cases} v_0 & \text{for } \pi/2 < \theta < \pi, \\ 0 & \text{otherwise.} \end{cases} \tag{10}$$

Although this is not an existing prediction for the system of Pt-PS colloid, we believe its comparison with the model that is based on current loops will be helpful. The velocity profiles $u_x(x, y = 0, z = 0)$ and $u_y(x = 0, y, z = 0)$ can be calculated and fitted to the experimental measurements using v_0 as a fit parameter (Supplementary Figure 5). The results are commented in the main text.

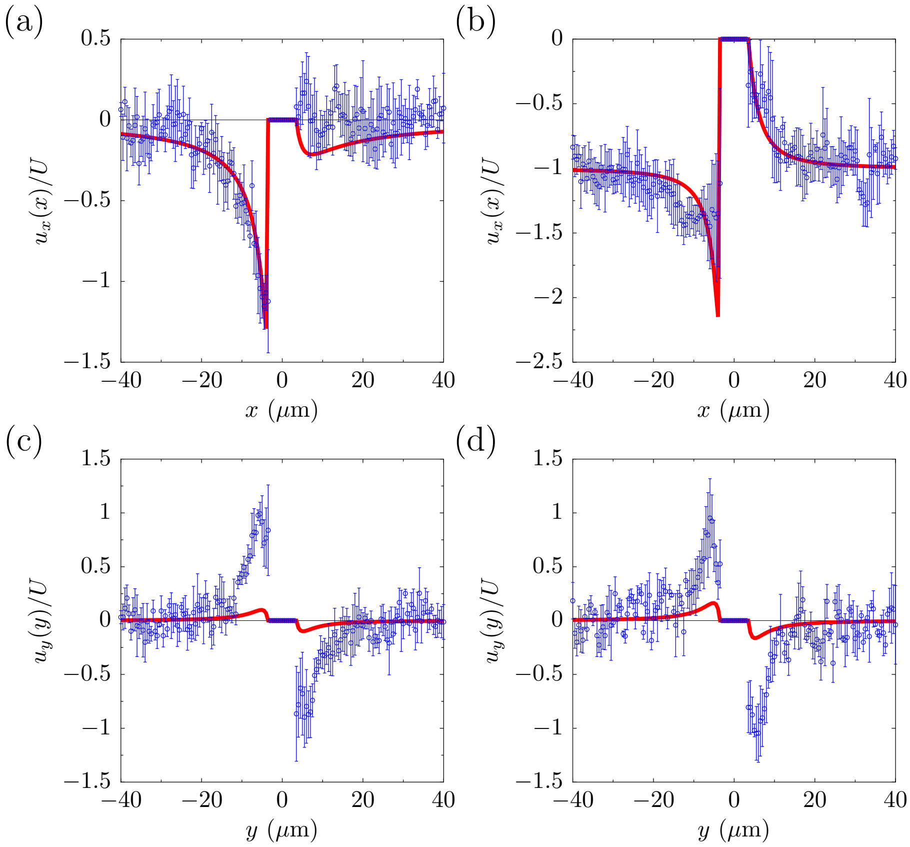

Supplementary Figure 5: Top: x-component of the velocity measured at $y = 0$ and as a function of the coordinate x for the two situations where the swimmer is stuck (a) and freely moving (b). Bottom: y-component of the velocity measured at $x = 0$ and as a function of the coordinate y for the two situations where the swimmer is stuck (c) and freely moving (d). The red line is a fit of the experimental data using constant slip velocity over the Pt hemisphere [Eq. (10)]. The values of the fit parameters are $K_{\text{stuck}} = 1.55 \pm 0.14$ and $K_{\text{moving}} = 2.55 \pm 0.21$.

Supplementary References

[1] S. E. Spagnolie, E. Lauga, Hydrodynamics of self-propulsion near a boundary: predictions and accuracy of far-field approximations, *J. Fluid Mech.* **700**, 105 (2012).

[2] K. Ishimoto, E. A. Gaffney, Squirmer dynamics near a boundary, *Phys. Rev. E* **88**, 0262702 (2013).

[3] J. R. Blake, A note on the image system for a stokeslet in a no-slip boundary, *Math. Proc. Cambridge* **70**, 303 (1971).
